# Antibacterial, Antioxidant, and Anti-Inflammatory Activities of Thai Stingless Bee Propolis Extracts

**DOI:** 10.3390/biology15171504

**Published:** 2026-09-02

**Authors:** Varachaya Intachaisri, Sureeporn Suriyaprom, Pornpimon Ngamsaard, Nitsanat Cheepchirasuk, Thida Kaewkod, Supakit Khacha-ananda, Yingmanee Tragoolpua

**Affiliations:** 1 Department of Biology, Faculty of Science, Chiang Mai University, Chiang Mai 50200, Thailand; varachaya.int@cmu.ac.th (V.I.); sureeporn.suriyaprom@cmu.ac.th (S.S.); promjinnapimon@gmail.com (P.N.); nitsanat_cheep@cmu.ac.th (N.C.); thida.kaewkod@cmu.ac.th (T.K.); 2 Office of Research Administration, Chiang Mai University, Chiang Mai 50200, Thailand; 3 Department of Forensic Medicine, Faculty of Medicine, Chiang Mai University, Chiang Mai 50200, Thailand; supakit.kh@cmu.ac.th

**Keywords:** bioactive compounds, antioxidant activity, antibacterial activity, anti-inflammatory activity, flavonoids, liquid chromatography–mass spectrometry, propolis, Thai stingless bee

## Abstract

Propolis is a resinous natural product collected by bees from plant sources to protect the hive. Its chemical composition varies according to bee species and environmental conditions, resulting in differences in its biological activities. This study aimed to evaluate the biological properties of stingless bee propolis collected from different provinces in Thailand, with particular attention to two native species, *Tetragonula pegdeni* and *Tetragonula laeviceps*. These two species of *Tetragonula* are commonly found in tropical regions of the country. The investigation focused on their antioxidant, antimicrobial, and anti-inflammatory activities. The propolis extract from *T*. *laeviceps* stingless bee (sample D) exhibited the highest total phenolic and flavonoid contents, at 29.59 mg GAE/g extract and 73.95 mg QUE/g extract, respectively. Among the propolis extracts, *T. pegdeni* stingless bee propolis extract (sample A) showed the greatest antioxidant activity of 7.08 ± 0.18 GAE/g extract and 75.64 ± 3.07 mg TEAC/g extract by the DPPH and ABTS assays, respectively, together with the highest FRAP value of 48.54 ± 0.48 mg FeSO_4_/g extract. The antibacterial efficacy of these extracts was further evaluated against skin pathogenic bacteria, including *Staphylococcus aureus*, *Staphylococcus epidermidis*, methicillin-resistant *Staphylococcus aureus* (MRSA), *Pseudomonas aeruginosa*, *Micrococcus luteus*, and *Cutibacterium acnes*. Inhibition zones ranged from 7.00 to 14.00 mm when determined by the agar disc diffusion method. Moreover, antibacterial activity was investigated by broth dilution assay, and MIC and MBC values ranged from 0.97 to 125 mg/mL. The cytotoxicity test of propolis extracts using RAW264.7 macrophage cells showed cell viability above 70%, and the selected concentrations were used for further anti-inflammatory evaluation. *T. pegdeni* stingless bee propolis extracts (samples A and B) demonstrated anti-inflammatory activity by reducing NO production and decreasing the expression of *iNOS*, *COX-2*, and *IL-1β* genes in LPS-stimulated RAW264.7 macrophage cells. LC–MS analysis revealed differences in chemical profiles among the propolis extracts and various bioactive compounds, including phenolic acids, flavonoids, xanthones, terpenoids, triterpenoids, and curcuminoids were identified. Therefore, Thai stingless bee propolis demonstrated potential as a natural source of bioactive compounds with antioxidant, antibacterial, and anti-inflammatory properties.

## Simple Summary

Skin infections and inflammation are common health problems that can affect quality of life and become more difficult to treat because of increasing antibiotic resistance. Therefore, there is growing interest in finding safe and effective natural products that may help prevent or reduce these conditions. Propolis, a natural substance produced by stingless bees from plant resins, has long been used in traditional medicine because of its health-promoting properties. Stingless bee propolis samples were collected from different regions in Thailand and investigated for their chemical composition, antioxidant, antibacterial, and anti-inflammatory activities. The results showed that the propolis extracts contained a range of compounds, including flavonoids, phenolic acids, xanthones and terpenoids identified by LC–MS analysis. The propolis extracts showed antioxidant activity in several assays, inhibited the growth of several bacteria associated with skin infections, and reduced nitric oxide production in stimulated macrophage cells, indicating anti-inflammatory potential. The antibacterial activity varied among individual propolis samples. Overall, the findings suggest that Thai stingless bee propolis is a promising natural resource for developing products that may help prevent skin infections, reduce inflammation, and support better skin health.

## 1. Introduction

Bees play a vital role in maintaining terrestrial ecosystems through plant pollination. Among them, stingless bees (Apidae: Meliponini) are eusocial insects that live in colonies and perform essential ecological functions [1]. They represent a diverse group of social bees, comprising more than 500 species distributed across tropical and subtropical regions [2]. In Thailand, 31 species from 10 genera have been documented, with *Tetragonula pegdeni* and *Tetragonula laeviceps* being the most commonly cultivated by local beekeepers [3]. *T. laeviceps* is widely distributed in Thailand and is commonly used in Thai meliponiculture. It exhibits opportunistic nest-site selection and can utilize cavities in human-made structures [4]. However, in Eastern Thailand, *T. pagdeni* has been reported to forage on a diverse range of floral resources, including cultivated plants and weeds, particularly during periods of limited floral resources [5]. In addition, these species may produce propolis with distinct chemical profiles, potentially leading to differences in their biological activities. These stingless bees produce and store various natural products, including honey, pollen, wax, and propolis, also known as cerumen [4]. Among these, propolis has attracted considerable scientific attention due to its diverse biological and pharmacological potential [5].

Propolis is a resinous material collected by bees from plant exudates, mixed with wax and mandibular secretions, and used to protect the hive from physical damage and microbial invasion. Propolis is a natural source of numerous bioactive compounds and has been reported to possess a broad range of pharmacological properties, including antimicrobial, antioxidant, anti-inflammatory, and anticancer activities [6,7,8,9]. Moreover, Thai stingless bee propolis from *T. laeviceps* and *Tetrigona melanoleuca* contains diverse bioactive compounds, including xanthones, triterpenes and lignans and demonstrates antibacterial activity [10]. In addition, propolis from *T. laeviceps* stingless bee exhibits antimicrobial activity against several bacteria and fungi, and antiproliferative activity against a colon carcinoma cell line [10].

In this context, skin and soft-tissue infections (SSTIs) represent a common type of bacterial infection worldwide and represent a major cause of medical consultation. These infections can result in serious complications, including hospitalization, surgical intervention, bacteremia, and even death, posing significant diagnostic and therapeutic challenges [11,12]. Most SSTIs are caused by Gram-positive bacteria, particularly *Staphylococcus aureus* and *Streptococcus pyogenes*, while opportunistic pathogens such as *Staphylococcus epidermidis*, *Cutibacterium acnes*, and *Pseudomonas aeruginosa* are also frequently involved [13]. In addition to microbial invasion, the inflammatory response plays a crucial role in the pathophysiology of SSTIs [14,15,16]. During infection, the host immune system activates the expression of inflammation-related genes and releases various pro-inflammatory mediators to eliminate invading bacteria [17,18]. However, excessive or prolonged inflammation can cause tissue damage, delay wound healing, and contribute to chronic skin disorders. Given the increasing concerns over bacterial resistance and inflammation-associated complications, there is a growing need for natural therapeutic agents that exhibit both antimicrobial and anti-inflammatory properties [19].

Furthermore, propolis has been widely reported to exert strong anti-inflammatory effects by suppressing the production and release of pro-inflammatory cytokines, thereby reducing excessive inflammatory responses. In addition, propolis is well-documented and has demonstrated antimicrobial potential. These properties suggest that stingless bee propolis could serve as a promising natural therapeutic agent for managing skin infections and inflammation-related skin conditions [20,21,22,23,24].

However, studies by investigating the antibacterial, antioxidant, and anti-inflammatory properties of stingless bee propolis, particularly from *Tetragonula* species in Thailand, remain limited. Therefore, this study aimed to evaluate the antimicrobial activity of stingless bee propolis extracts against common skin pathogenic bacteria, as well as to assess their antioxidant and anti-inflammatory potentials.

## 2. Materials and Methods

### 2.1. Sample Collection and Preparation of Propolis

Propolis samples produced by *Tetragonula pegdeni* and *Tetragonula laeviceps* stingless bees were collected during the rainy season in August 2023 from local stingless bee farms in Chumphon, Chiang Mai, and Sa Kaeo Provinces, Thailand. Detailed information on the sampling locations, geographic coordinates, and surrounding environments is provided in Table 1. The collected samples were cut into small pieces and stored at 4 °C to preserve their chemical integrity until extraction.

### 2.2. Propolis Extraction

The propolis samples from *T. pegdeni* and *T. laeviceps* stingless bees were macerated in 70% ethanol for extraction at a ratio of 1:20 (*w*/*v*; propolis to solvent). The mixture was shaken at 150 rpm on an orbital shaker (Gallenkamp, London, UK) for 72 h at room temperature and subsequently filtered through Whatman No. 1 filter paper (Maidstone, Kent, UK) to remove insoluble materials, and the solvent was then evaporated under reduced pressure using a rotary evaporator (Heidolph, Schwabach, Germany) at 45 °C to obtain the crude ethanolic extract. The extract was subsequently freeze-dried using a lyophilizer (LABCONCO, Kansas City, MO, USA) to yield the dried propolis extract. The propolis extract was kept at 4 °C until further analysis [25].

### 2.3. Total Phenolic Content Analysis of Propolis Extracts

Total phenolic content was measured in the propolis extracts using the Folin–Ciocalteu colorimetric method [26]. The propolis extract was diluted in methanol before analysis. In brief, 25 μL of the diluted sample was combined with 12.5 μL of 50% Folin–Ciocalteu reagent (Billerica, MA, USA), 125 μL of distilled water, 25 μL of 95% ethanol, and 25 μL of 5% sodium carbonate in a 96-well microplate. The reaction mixture was incubated in the dark at room temperature for 1 h, after which the absorbance was recorded at 725 nm using a microplate reader (DYNEX Technologies, Chantilly, VA, USA). Gallic acid (Sigma-Aldrich, St. Louis, MO, USA) (0.01–0.25 mg/mL) was used to perform the calibration curve. Total phenolic content was reported as milligrams of gallic acid equivalents per gram of extract (mg GAE/g extract).

### 2.4. Total Flavonoid Content Analysis of Propolis Extracts

Total flavonoid content was measured in the propolis extracts by the aluminum chloride colorimetric method using quercetin (Billerica, MA, USA) as a standard compound [27]. The quercetin stock solution was prepared in ethanol and diluted to generate the standard curve. The propolis extract was prepared in ethanol at a suitable concentration for analysis. For the assay, 20 µL of the standard or sample solution was mixed with 4 µL of 10% (*w*/*v*) aluminum chloride (Sigma-Aldrich, St. Louis, MO, USA), 60 µL of methanol (RCI LABSCAN Limited, Bangkok, Thailand), 4 µL of 1 M CH_3_COOK (Daejung Chemicals, Siheung, Republic of Korea), and 112 µL of deionized water. Then, the mixtures were incubated at room temperature for 30 min, and the absorbance was measured at 415 nm using a microplate reader (DYNEX Technologies, Chantilly, VA, USA). The total flavonoid content was calculated and reported as milligrams of quercetin equivalent per g of extract (mg QE/g extract).

### 2.5. Chemical Composition Analysis of Propolis Extract by LC–MS/MS

The phytochemical composition of the propolis extracts was analyzed using liquid chromatography coupled with tandem mass spectrometry (LC–MS/MS). Propolis samples were dissolved in 70% methanol containing sulfadimethoxine (50 ng/mL) as an internal standard to obtain a final concentration of 10 mg/mL. After centrifugation at 14,000 rpm for 10 min, the supernatants were collected and subjected to LC–MS analysis.

Chromatographic separation was performed on a Poroshell 120 EC-C18 column (2.1 × 100 mm, 2.7 µm; Agilent Technologies, Santa Clara, CA, USA) maintained at 50 °C. The mobile phase consisted of 0.1% formic acid (Merck, Darmstadt, Germany) in water (A) and 0.1% formic acid in acetonitrile (B), delivered at a flow rate of 0.4 mL/min. The injection volume was 10 µL. The gradient elution programme is presented in Table 2.

Mass spectrometric analysis was performed using an Agilent 6545XT AdvanceBio QTOF LC-MS system (Agilent Technologies, Santa Clara, CA, USA) equipped with an electrospray ionization source and operated in both positive and negative ionization modes. The drying gas temperature was set at 325 °C with a flow rate of 13 L/h, while the sheath gas temperature was set at 275 °C with a flow rate of 12 L/h. The nebulizer pressure was 45 psi. The capillary voltage was set to 4000 V in positive mode and 3000 V in negative mode. MS1 data were acquired over a mass range of *m*/*z* 40–1700, while MS2 data were acquired over a mass range of *m*/*z* 25–1000. The collision energy was set to 20 eV in positive mode and 10 eV in negative mode.

Data were acquired at a rate of 3.35 spectra/s, with up to 10 precursor ions selected per cycle for fragmentation using a precursor ion threshold of 5000 counts. Continuous internal mass calibration was performed using reference masses at *m*/*z* 121.0509 and 922.0098 in positive mode and *m*/*z* 112.9856 and 1033.9881 in negative mode. The LC–MS/MS analytical conditions were consistent with those previously reported using the Agilent 6545XT AdvanceBio QTOF platform (Agilent Technologies, Santa Clara, CA, USA) and a Poroshell 120 EC-C18 column (Agilent Technologies, Santa Clara, CA, USA) [28].

### 2.6. DPPH Radical Scavenging Activity of Propolis Extracts

The antioxidant activity of the propolis extract was evaluated using the DPPH radical scavenging assay. A total of 50 µL of the extract was diluted and mixed with 150 µL of DPPH (Sigma-Aldrich, St. Louis, MO, USA) working solution in a 96-well microplate. The mixtures were incubated at room temperature in the dark, and the absorbance was subsequently measured at 517 nm using a microplate reader. Methanol was used as the blank, while a DPPH solution without extract served as the control.

Antioxidant capacity was determined using a gallic acid calibration curve within a range of 0.001 to 0.01 mg/mL (R^2^ = 0.98) and expressed as milligrams of gallic acid equivalents per gram of extract (mg GAE/g extract) [29]. The IC_50_ values were calculated by linear regression using Microsoft Excel. The concentration and radical-scavenging activity were plotted to obtain the linear equation (*y = mx + c*), and the IC_50_ value was calculated by setting *y* to 50%.

### 2.7. ABTS Radical Scavenging Assay of Propolis Extracts

The antioxidant activity of the propolis extracts was also evaluated using the ABTS radical cation decolorization assay. The ABTS solution (Sigma-Aldrich, St. Louis, MO, USA) was prepared by mixing 7 mM ABTS diammonium salt with 2.45 mM potassium persulfate (Sigma-Aldrich, St. Louis, MO, USA) and incubating the mixture in the dark at room temperature for 12–16 h. Before the assay, the ABTS solution was diluted with distilled water to achieve an absorbance of 0.700 ± 0.020 at 734 nm. For the assay, 5 μL of the propolis extract was mixed with 195 μL of the diluted ABTS solution in a 96-well microplate and incubated in the dark at room temperature for 10 min. The absorbance was then measured at 734 nm using a microplate reader. Trolox (Sigma-Aldrich, St. Louis, MO, USA) was used as the antioxidant standard within a range of 0.025 to 0.45 mg/mL (R^2^ = 0.98), and the antioxidant capacity was expressed as milligrams of Trolox equivalents per gram of extract (mg TE/g extract) [30]. The IC_50_ values were calculated by linear regression using Microsoft Excel. The concentration and radical-scavenging activity were plotted to obtain the linear equation (*y = mx + c*), and the IC_50_ value was calculated by setting *y* to 50%.

### 2.8. Ferric Reducing Antioxidant Power Assay of Propolis Extracts

The ferric reducing antioxidant power of the propolis extracts was assessed using the FRAP assay [31]. The working reagent was freshly prepared by combining 300 mM acetate buffer (RCI LABSCAN Limited, Bangkok, Thailand) (pH 3.6), 10 mM TPTZ (Merck, Darmstadt, Germany) (2,4,6-tri(2-pyridyl)-s-triazine) in 40 mM HCl (RCI LABSCAN Limited, Bangkok, Thailand), and 20 mM ferric chloride (Sigma-Aldrich, St. Louis, MO, USA) solution at a volume ratio of 10:1:1. A total of 50 μL of each extract was mixed with 150 μL of FRAP reagent (Merck, Darmstadt, Germany). The reaction mixture was incubated at room temperature for 15 min, and the absorbance was then recorded at 593 nm using microplate reader. Antioxidant capacity was determined from a ferrous sulphate (FeSO_4_) (Sigma-Aldrich, St. Louis, MO, USA) standard curve and reported as mg FeSO_4_ equivalents per gram of extract (mg FeSO_4_/g extract).

### 2.9. Antibacterial Activities of Propolis Extracts

#### 2.9.1. Bacterial Strains

The skin pathogenic bacteria included in this study were *Staphylococcus aureus* ATCC 25923, methicillin-resistant *S*. *aureus* (MRSA) 49, *S*. *epidermidis* ATCC 14990, *Pseudomonas aeruginosa* ATCC 27853, *Micrococcus luteus*, and *Cutibacterium acnes* DMST 14916. The bacterial strains were obtained from the SCB 2711 Microbiology Laboratory and used to evaluate the antibacterial activity of the propolis extracts.

#### 2.9.2. Agar Disc Diffusion Method

The antibacterial activity of the propolis extracts was evaluated by the disc diffusion method [23]. Bacterial suspensions were adjusted to a turbidity equivalent to 0.5 McFarland (approximately 1 × 10^8^ CFU/mL) and swabbed onto Mueller–Hinton agar (MHA) (Becton, Dickinson and Company, Sparks, MD, USA), except for *C. acnes*, which was plated on BHI agar (Becton, Dickinson and Company, Sparks, MD, USA).

The propolis extract was prepared in DMSO (RCI LABSCAN Limited, Bangkok, Thailand) at a final concentration of 500 mg/mL. Sterile paper discs, 6 mm in diameter (MACHEREY-NAGEL (MN) 827, Düren, Germany) were impregnated with the extract and placed on the inoculated agar surface. The loading capacity of the disc was 20 µL, and the use of MN 827 antibiotic test disc (6 mm) discs for antimicrobial assays has also been reported previously [32]. The antibacterial assays were performed in triplicate.

Gentamicin was served as the positive control, while 100% DMSO was used as the negative control. All bacterial strains except *C. acnes* were incubated at 37 °C for 24 h under aerobic conditions. However, *C. acnes* was cultured at 37 °C for 24 h under anaerobic conditions. Antibacterial activity was determined by measuring the diameters of the inhibition zones after incubation.

#### 2.9.3. Minimum Inhibitory Concentration (MIC) and Minimum Bactericidal Concentration (MBC)

The minimum inhibitory concentration (MIC) of propolis extract was determined using the broth dilution method [23,33]. A stock solution of propolis extract (500 mg/mL) was prepared in Mueller–Hinton broth (MHB) and serially two-fold diluted in a sterile 96-well microtiter plate, except for *C*. *acnes*, which was diluted in BHI broth. Bacterial suspensions cultured for 24 h (or 72 h for *C*. *acnes*) were adjusted to a turbidity of 0.5 McFarland and added to each well. The plates were incubated at 37 °C for 24 h or 72 h for *C*. *acnes*. The MBC was determined by streaking a plate of treated bacterial culture from wells showing no visible bacterial growth in the MIC assay onto agar plates and evaluating the presence or absence of bacterial growth after incubation. The MBC was defined as the lowest concentration of propolis extract that showed no visible bacterial growth on the agar plates [34].

### 2.10. Cell Culture

RAW 264.7 macrophage cell line originated from mice (*Mus musculus*) and was purchased from ATCC (Manassas, VA, USA) with the accession number ATCC-TIB-71. Cells were maintained in Dulbecco’s modified Eagle’s medium (DMEM) (Gibco, Grand Island, NY, USA). supplemented with 10% fetal bovine serum (FBS) (Gibco, Grand Island, NY, USA), 100 units/mL penicillin (Gibco, Grand Island, NY, USA), and 100 µg/mL streptomycin (Gibco, Grand Island, NY, USA). Cells were maintained in a humidified 5% CO_2_ incubator (SHEL LAB, Cornelius, OR, USA) at 37 °C, with the culture medium replaced every 2–3 days [35]. A subculture of the confluent cells was performed once the cells achieved 80–90% confluency for further experiments.

### 2.11. Cell Toxicity Test

The cytotoxicity of the propolis extracts was assessed using the MTT (3-(4,5-dimethylthiazol-2-yl)-2,5-diphenyl tetrazolium bromide) (Bio Basic, Toronto, ON, Canada) assay. RAW 264.7 cells were seeded in a 96-well plate (1 × 10 ^6^ cells/well) and incubated at 37 °C with 5% CO_2_ for 24 h. The culture medium was then removed, and the cells were treated with 100 µL of control medium or medium containing different concentrations of propolis extract for 24 h. After treatment, 50 µL of the MTT reagent (2 mg/mL) was added to each well and incubated for 4 h to allow formazan crystal formation. Following incubation, the medium was removed, and the formed formazan crystals were dissolved in DMSO. The absorbance was then recorded at 540 nm using a reference wavelength of 630 nm with a microplate reader.

### 2.12. Inhibitory Effects of Propolis Extracts on Nitric Oxide (NO) Production from RAW Macrophage 264.7 Cells

#### 2.12.1. Treatment of RAW Macrophage 264.7 Cells with Propolis Extract Before Stimulation with LPS

RAW 264.7 cells were seeded into a 96-well microplate at a density of 1 × 10^6^ cells/mL and incubated at 37 °C in a humidified atmosphere containing 5% CO_2_ for 24 h. The culture medium was then removed, and the cells were treated with propolis extracts at concentrations of 0.078–0.312 mg/mL or L-NAME (Sigma-Aldrich, St. Louis, MO, USA) at concentrations of 0.312–10 mg/mL for 1 h. LPS (Sigma-Aldrich, St. Louis, MO, USA) was then added to a final concentration of 1 µg/mL, and the cells were incubated for a further 24 h. The culture supernatant was collected for NO determination.

#### 2.12.2. Treatment of RAW Macrophage 264.7 Cells by Propolis Extract After Stimulation with LPS

RAW 264.7 cells were seeded into a 96-well microplate at a density of 1 × 10^6^ cells/mL and incubated at 37 °C in a humidified atmosphere containing 5% CO_2_ for 24 h. The cells were first stimulated with LPS at a final concentration of 1 µg/mL for 10 min. Then, the LPS-containing medium was removed, and the cells were treated with propolis extracts at concentrations of 0.078–0.312 mg/mL or L-NAME at concentrations of 0.312–10 mg/mL for 24 h. The culture supernatant was collected for NO determination.

#### 2.12.3. Measurement of NO Production

NO production was determined by measuring nitrite accumulation in the culture supernatant using the Griess reagent. Briefly, 100 µL of culture supernatant was mixed with 100 µL of Griess reagent (Sigma-Aldrich, St. Louis, MO, USA), consisting of 1% (*w*/*v*) sulfanilamide in 5% (*v*/*v*) phosphoric acid and 0.1% (*w*/*v*) naphthylethylenediamine dihydrochloride. The mixture was incubated at room temperature for 15 min, and the absorbance was measured at 550 nm using a microplate reader. Nitrite concentrations were calculated from a sodium nitrite standard curve [35,36].

### 2.13. Inhibitory Effects on Inflammatory Gene Expression

The mRNA expression levels of inflammatory-related genes, including *iNOS*, *COX-2*, and *IL-1β*, were evaluated following treatment with propolis extract using quantitative real-time PCR (qRT-PCR). RAW 264.7 cells were stimulated with 1 μg/mL lipopolysaccharide (LPS) for 10 min, followed by post-treatment with the propolis samples for 24 h. Total RNA was extracted from RAW 264.7 cells using TRIzol™ reagent (Invitrogen, Carlsbad, CA, USA) according to the manufacturer’s instructions. RNA purity was assessed using a NanoDrop spectrophotometer (Thermo Scientific, Waltham, MA, USA). Samples with A260/A280 ratios ranging from 1.8 to 2.0 were considered suitable for further analysis. For cDNA synthesis, 1000 ng of total RNA was reverse-transcribed using ReverTra Ace qPCR RT Master Mix (TOYOBO, Osaka, Japan). Quantitative PCR was performed using 100 ng of cDNA, 2× SensiFAST SYBR No-ROX Mix (BIOLINE, London, UK), and 400 nM each of forward and reverse primers. A control without template was included in each run to verify the absence of contamination. Amplification and data acquisition were carried out using a qTOWER3 Real-Time PCR system (Analytik Jena, Jena, Germany). The relative gene expression levels were normalized to β-actin as the internal control and calculated using the 2^−^^ΔΔCt^ method [36].

### 2.14. Statistical Analysis

All quantitative experiments were conducted in triplicate, and the data are presented as mean ± standard deviation (SD). Differences among groups were analyzed using one-way analysis of variance (ANOVA), followed by Tukey’s honestly significant difference (HSD) test for multiple comparisons. Statistical significance was defined as a *p*-value < 0.05. All analyses were performed using SPSS software (version 17.0, IBM Corp., Chicago, IL, USA). The bar graphs and statistical analyses were generated using Prism 10 (GraphPad Software, Inc., La Jolla, CA, USA).

## 3. Results

### 3.1. Extraction Yield and Physical Characteristics of Propolis Extracts

Propolis samples from *T. pegdeni* and *T. laeviceps* stingless bees were extracted with 70% ethanol at a propolis-to-solvent ratio of 1:20 (*w*/*v*) using the maceration technique for 72 h. Then, the solvent was removed under reduced pressure, and the propolis extracts were lyophilized to obtain the dried extract. The highest extraction yield of 28.51% was obtained after extraction of propolis from *T*. *laeviceps* (sample D), followed by the *T*. *pegdeni* samples C, B, and A, with extraction yields of 25.77%, 21.97%, and 18.63%, respectively (Table 3).

**Figure 1:**
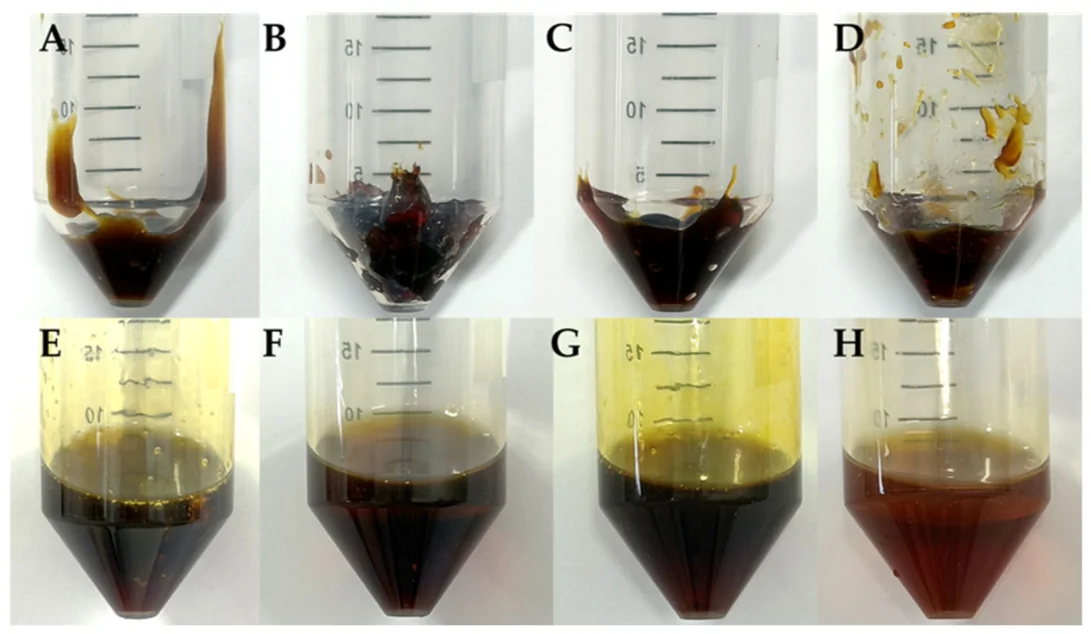
Physical appearance of propolis extracts before and after dissolution in DMSO. Samples A–D are shown before dissolution (**A**–**D**), while the corresponding dissolved samples A–D are shown after dissolution in DMSO (**E**–**H**), respectively.

Before dissolving, the propolis extracts appeared as sticky resinous materials with colours ranging from dark brown to light brown (Figure 1A–D). After dissolving in DMSO, all extracts formed homogeneous brown solutions, and their colours remained consistent, ranging from dark brown to light brown. The resulting solutions were clear to slightly turbid, with no visible precipitation (Figure 1E–H).

### 3.2. Total Phenolic and Flavonoid Contents in Propolis Extracts

The total phenolic content (TPC) and total flavonoid content (TFC) of the propolis extracts obtained from two stingless bee species were determined using the Folin–Ciocalteu colorimetric method and the aluminum chloride colorimetric assay, respectively. As shown in Table 4, the propolis extract from *T. laeviceps* (sample D) exhibited the highest levels of both phenolic and flavonoid compounds, with values of 29.59 ± 0.28 mg GAE/g extract and 73.95 ± 4.62 mg QUE/g extract, respectively. Among the *T*. *pegdeni* extracts, sample A showed the highest total phenolic content (9.29 ± 0.25 mg GAE/g extract), whereas sample B exhibited the highest total flavonoid content (45.02 ± 1.50 mg QUE/g extract).

### 3.3. Phytochemical Compounds in Propolis Extract

**Figure 2:**
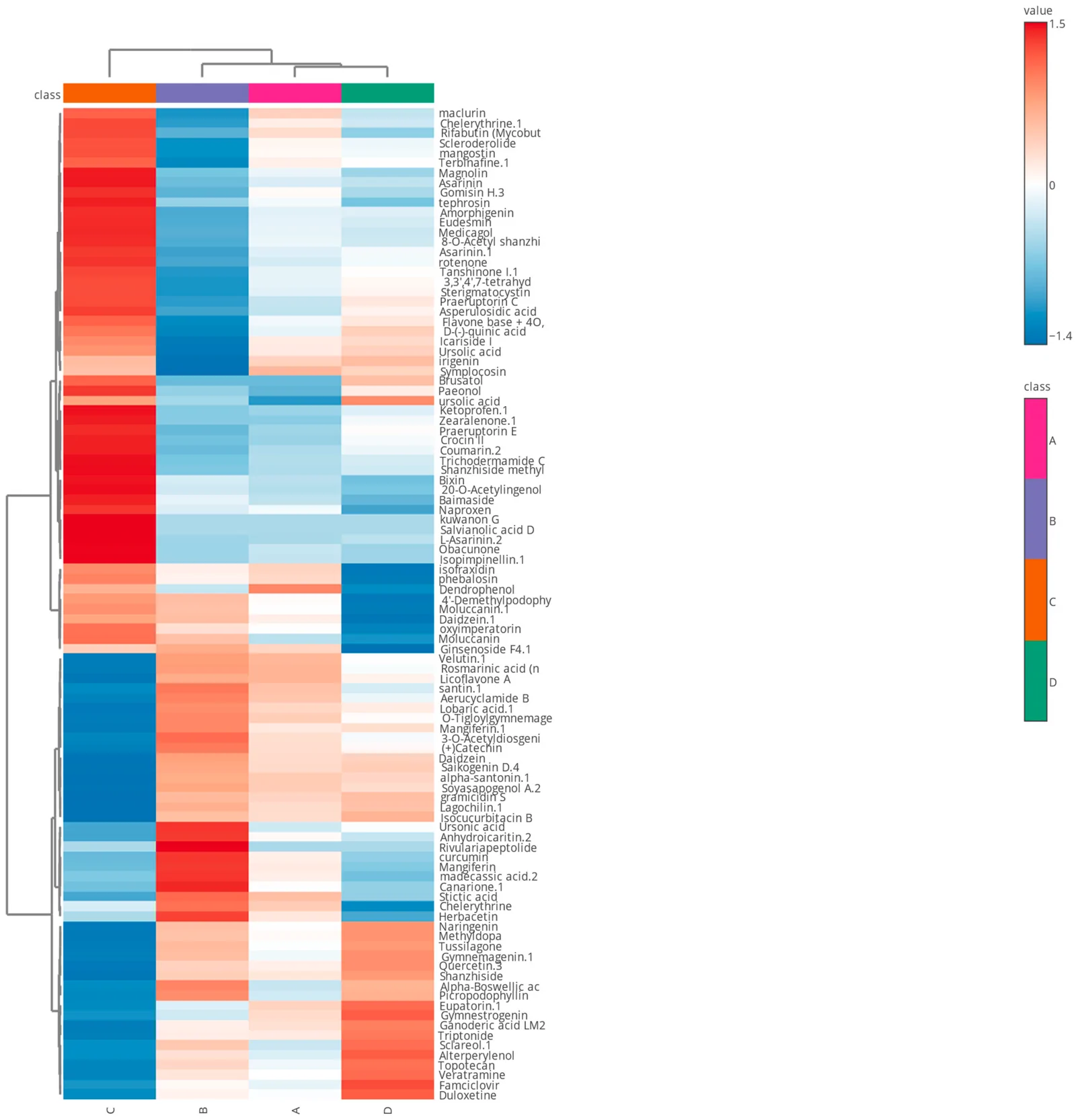
Heat map of tentatively identified metabolites in propolis extracts analyzed by LC–MS in positive ionization mode. Columns represent propolis samples (A–D), and rows represent the detected metabolites. Red and blue indicate higher and lower metabolite values, respectively. Hierarchical clustering was performed based on the metabolite profiles of the samples.

**Figure 3:**
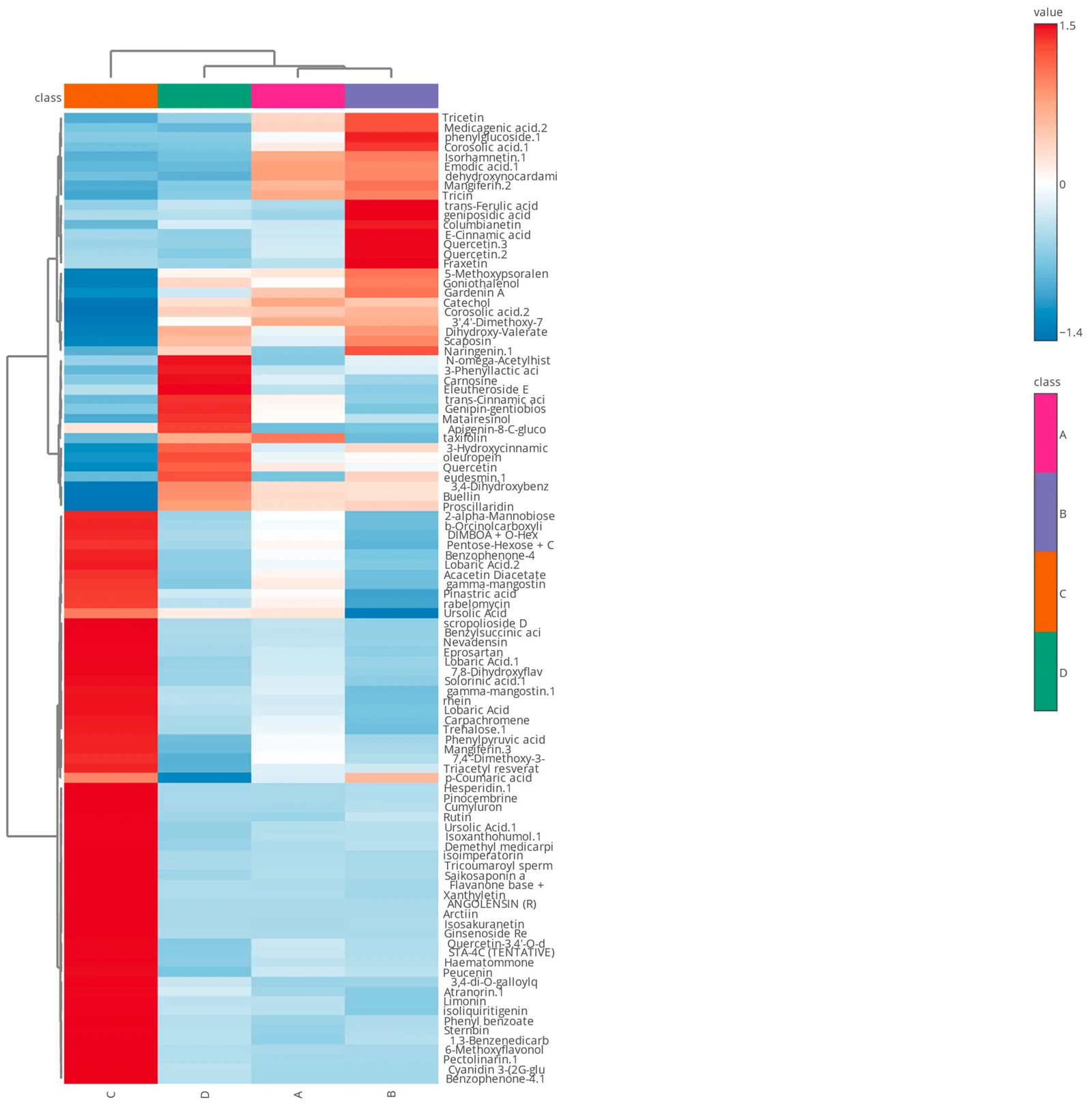
Heat map of tentatively identified metabolites in propolis extracts analyzed by LC–MS in negative ionization mode. Columns represent propolis samples (A–D), and rows represent the detected metabolites. Red and blue indicate higher and lower metabolite values, respectively. Hierarchical clustering was performed based on the metabolite profiles of the samples. The top colour bar denotes the sample groups.

LC–MS analysis was performed in both positive and negative ion modes to characterize the metabolite profiles of the propolis extracts. The heat maps (Figure 2 and Figure 3) showed that the metabolite profiles differed among the four propolis extracts. The major metabolite classes included flavonoids, phenolic acids, xanthones, terpenoids, and triterpenoids. These chemical classes have also been reported in stingless bee propolis from different sources [10,32]. Among the flavonoids identified were quercetin, acacetin, hesperidin, isoliquiritigenin, taxifolin, and pinocembrin. Curcumin, a curcuminoid, was also detected in the propolis extracts. In addition, trans-caffeic acid, rosmarinic acid, and *p*-coumaric acid were identified as phenolic acids, whereas mangiferin and corosolic acid were classified as a xanthone and a triterpenoid, respectively. The abundance of these metabolites varied among the propolis extracts. *T. pegdeni* stingless bee propolis extract (sample A) was characterized by the presence of rosmarinic acid, taxifolin, velutin, and licoflavone A. *T. pegdeni* stingless bee propolis extract (sample B) contained relatively higher levels of *p*-coumaric acid, quercetin, curcumin, and mangiferin. In contrast, *T. pegdeni* stingless bee propolis extract (sample C) was enriched in pinocembrin, α-mangostin, and coumarin, whereas quercetin was the predominant metabolite detected in *T*. *laeviceps* stingless bee propolis extract (sample D).

### 3.4. Antioxidant Activities of Propolis Extracts

The antioxidant activity of the propolis extracts was assessed using three complementary assays including DPPH radical scavenging, ABTS radical cation decolorization, and ferric reducing antioxidant power (FRAP). For the DPPH and ABTS assays, IC_50_ values were determined to indicate the concentration of extract required to achieve 50% radical scavenging. Among the tested extracts, sample A from *T. pegdeni* showed the highest antioxidant activity in the DPPH assay, with an IC_50_ value of 0.80 ± 0.02 mg/mL and an antioxidant capacity of 7.08 ± 0.18 mg GAE/g extract. *T. pegdeni* stingless bee propolis extract (sample C) showed the secondhighest antioxidant activity, while the remaining extracts demonstrated lower radical scavenging activities.

The ABTS assay showed a comparable pattern, with *T. pegdeni* stingless bee propolis extract (sample A) displaying the greatest antioxidant capacity, with an IC_50_ value of 3.64 ± 0.00 mg/mL and an antioxidant activity of 75.64 ± 3.07 mg TEAC/g extract, followed by *T. pegdeni* stingless bee propolis extract (sample C). The other extracts showed comparatively lower antioxidant activities (Table 5).

Similarly, *T. pegdeni* stingless bee propolis extract (sample A) showed the highest ferric reducing antioxidant capacity in the FRAP assay, with a value of 48.54 ± 0.48 mg FeSO_4_/g extract, followed by *T. laeviceps* stingless bee propolis extract (sample D) and *T. pegdeni* stingless bee propolis extract (sample C). *T. pegdeni* stingless bee propolis extract (sample B) demonstrated the lowest reducing activity (Table 5). Previous studies have also reported differences in antioxidant activity and phenolic and flavonoid contents among stingless bee propolis samples [37].

### 3.5. Antibacterial Activity of Propolis Extracts by Agar Disc Diffusion Assay

The antibacterial activities of propolis extracts obtained from two stingless bee species, *T*. *pegdeni* and *T*. *laeviceps*, against skin pathogenic bacteria, including *S*. *aureus*, *S*. *epidermidis*, methicillin-resistant *S*. *aureus* (MRSA), *Ps*. *aeruginosa*, *M*. *luteus*, and *C*. *acnes*, were evaluated using the agar disc diffusion method at a concentration of 500 mg/mL. Dimethyl sulfoxide (DMSO) was used as the negative control. Gentamicin was used as a positive control for the determination of antibacterial activity, except for MRSA. Vancomycin (1 mg/mL) was used as a positive control for MRSA.

**Figure 4:**
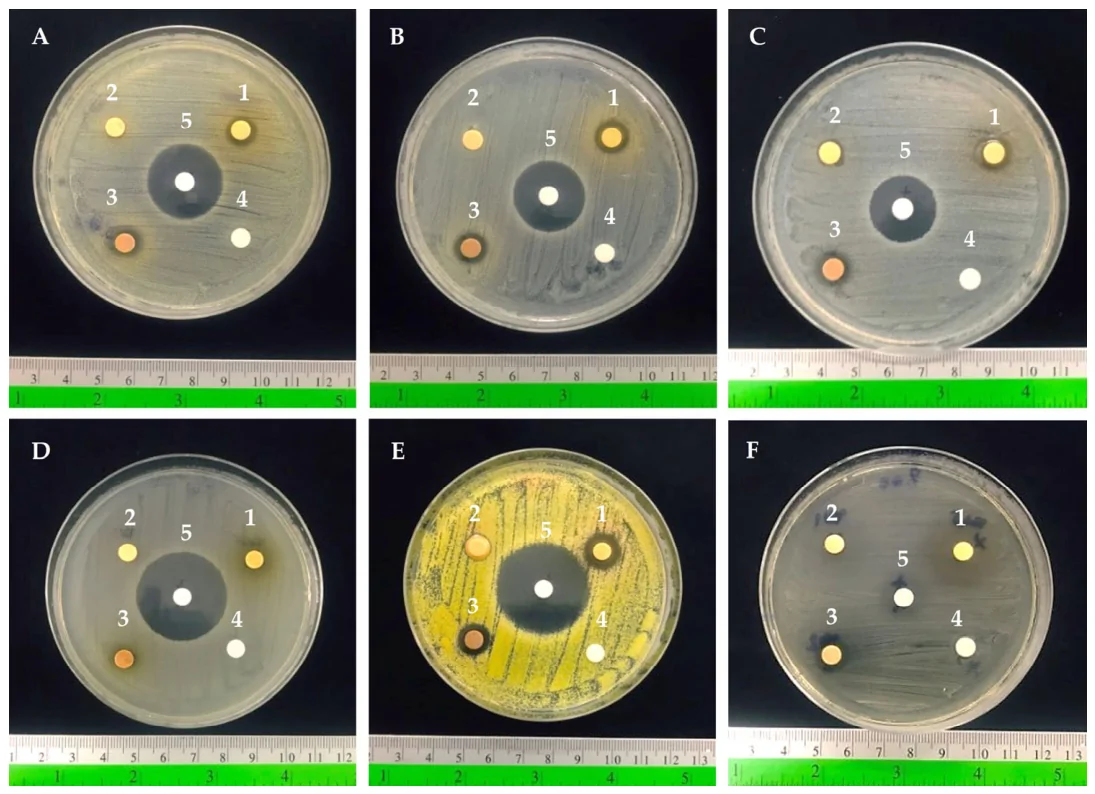
Antibacterial activity of *T. pegdeni* propolis extracts against skin pathogenic bacteria assessed using the agar disc diffusion assay. The tested samples were (1) Sample A, (2) Sample B, and (3) Sample C. DMSO (4) was used as the negative control, while gentamicin (1 mg/mL) (5) was used as the positive control. Vancomycin (1 mg/mL) was used as the positive control for MRSA. The tested bacterial strains were (**A**) *S. aureus*, (**B**) *S. epidermidis*, (**C**) MRSA, (**D**) *P. aeruginosa*, (**E**) *M. luteus*, and (**F**) *C. acnes*.

**Figure 5:**
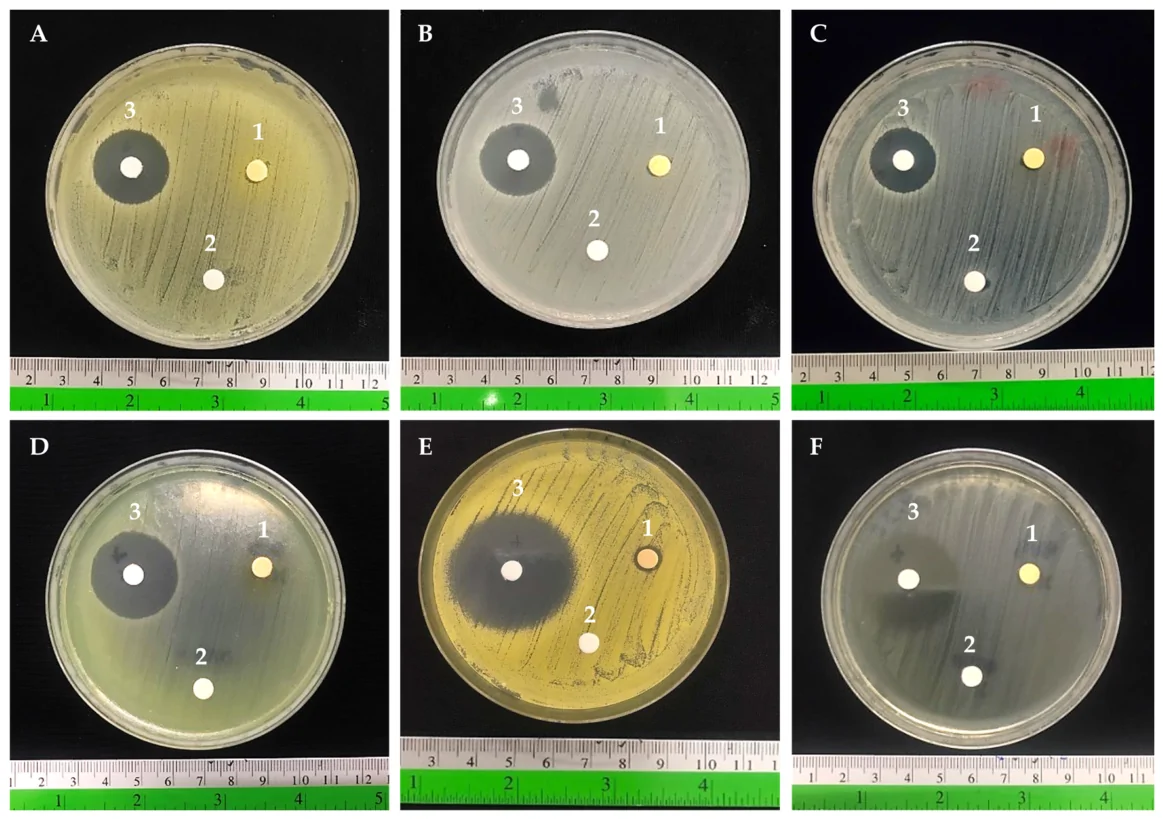
Antibacterial activity of *T. laeviceps* propolis extract against skin pathogenic bacteria assessed using the agar disc diffusion assay. The tested sample was (1) Sample D. DMSO (2) was used as the negative control, while gentamicin (1 mg/mL) (3) was used as the positive control. Vancomycin (1 mg/mL) was used as the positive control for MRSA. The tested bacterial strains were (**A**) *S. aureus*, (**B**) *S. epidermidis*, (**C**) MRSA, (**D**) *Ps*. *aeruginosa*, (**E**) *M*. *luteus*, and (**F**) *C*. *acnes*.

The *T*. *pegdeni* stingless bee propolis extract (sample A) exhibited the broadest antibacterial spectrum, showing inhibitory activity against all tested bacteria, with inhibition zone diameters ranging from 7.00 ± 0.00 to 14.00 ± 0.00 mm. Antibacterial activity of *T*. *pegdeni* stingless bee propolis extract (sample C) was also observed against several bacterial strains, including *S. aureus*, *S. epidermidis*, MRSA, *M. luteus*, and *C. acnes*, but showed no inhibitory effect against *Ps*. *aeruginosa*. *T*. *pegdeni* (sample C) exhibited the largest inhibition zone against *M*. *luteus*, with a diameter of 11.33 ± 0.58 mm. In contrast, *T*. *pegdeni* stingless bee propolis extract (sample B) could inhibit only MRSA, with an inhibition zone diameter of 7.83 ± 0.29 mm. Furthermore, the propolis extract from *T*. *laeviceps* (sample D) showed inhibitory activity only against *M*. *luteus*, with an inhibition zone diameter of 8.67 ± 0.58 mm (Table 6; Figure 4 and Figure 5).

### 3.6. Determination of Minimum Inhibitory Concentration (MIC) and Minimum Bactericidal Concentration (MBC)

The antibacterial activity of propolis extracts from two stingless bee species was evaluated by determining their minimum inhibitory concentrations (MICs) and minimum bactericidal concentrations (MBCs) using the broth dilution method. The propolis extracts were initially prepared at 500 mg/mL and subjected to two-fold serial dilution, whereas gentamicin and vancomycin (1 mg/mL) served as positive controls. The propolis extracts from *T*. *pegdeni* (samples A, B and C) exhibited strong antibacterial activity against *M*. *luteus*, with both MIC and MBC values of 1.95 mg/mL.

*T*. *pegdeni* stingless bee propolis extract (sample A) also demonstrated strong antibacterial activity against *S*. *aureus*, with MIC and MBC values of 3.90 mg/mL. In addition, *T*. *pegdeni* stingless bee propolis extract (sample B) showed broad-spectrum antibacterial activity against several Gram-positive bacteria, including *S*. *aureus*, *S*. *epidermidis*, MRSA, *M*. *luteus*, and *C*. *acnes*, with MIC and MBC values of 1.95 to 15.63 mg/mL.

*T*. *pegdeni* stingless bee propolis extract (sample C) showed antibacterial activity against *S*. *aureus*, *S*. *epidermidis*, *M*. *luteus*, and *C*. *acnes*, with MIC and MBC values ranging from 1.95 to 125 mg/mL. Furthermore, the propolis extract from *T*. *laeviceps* (sample D) showed the strongest antibacterial activity against *M*. *luteus*, with both MIC and MBC values of 0.97 mg/mL (Table 7).

### 3.7. Cell Viability Assessed by MTT Assay

**Figure 6:**
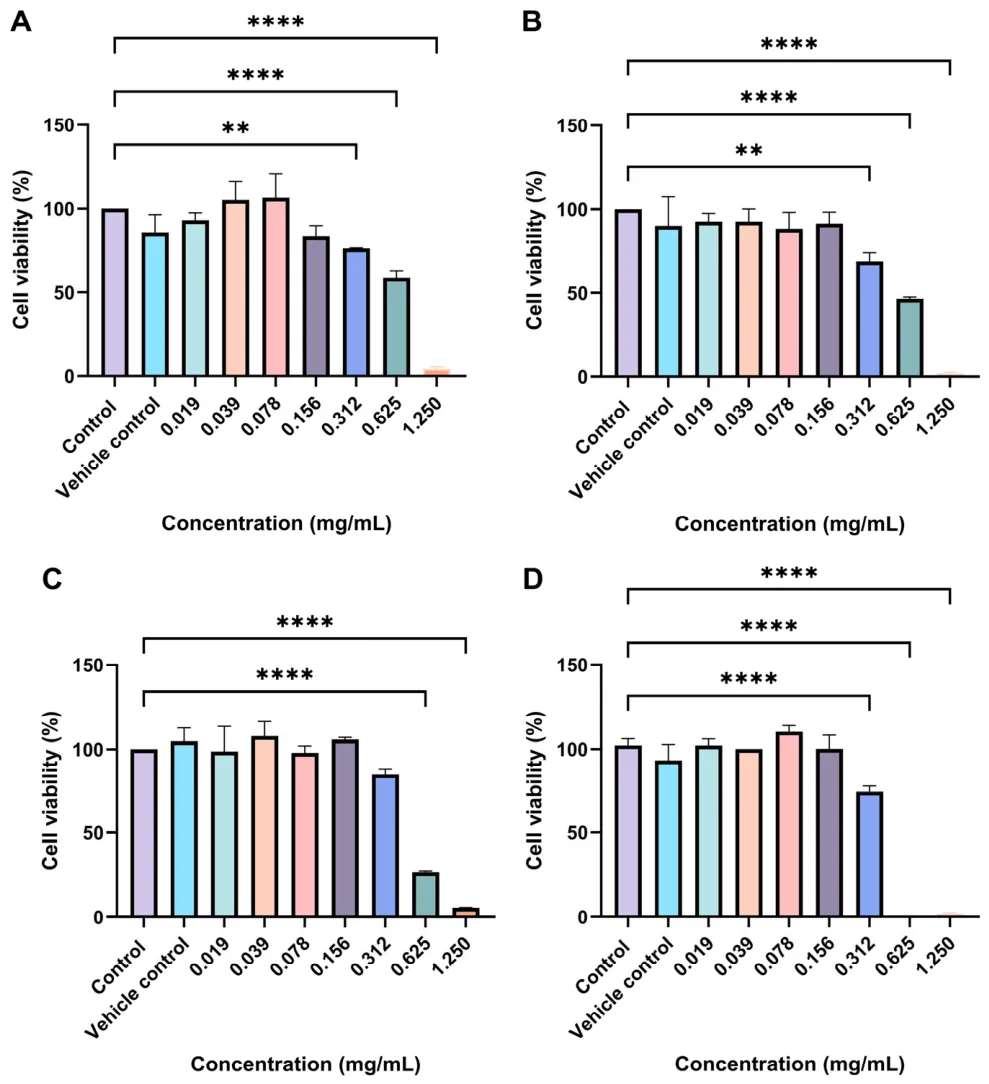
Cell viability of RAW264.7 macrophage cells after treatment with propolis extracts at concentrations ranging from 0.019 to 1.250 mg/mL for 24 h. (**A**) Sample A, (**B**) Sample B, (**C**) Sample C, and (**D**) Sample D. Asterisk indicates significant difference, ** (*p* ≤ 0.01), and **** (*p* ≤ 0.0001).

The cytotoxicity of propolis extracts was evaluated in RAW264.7 macrophages using the MTT assay at concentrations ranging from 0.019 to 1.25 mg/mL. All four propolis extracts exhibited cell viability greater than 70% at all tested concentrations compared with untreated control cells (Figure 6). In addition, 1% dimethyl sulfoxide (DMSO), used as the vehicle control, showed no cytotoxic effect on RAW264.7 cells. Based on the cell viability findings, the anti-inflammatory activity of the propolis extracts was further assessed at concentrations of 0.078–0.312 mg/mL by measuring nitric oxide production in LPS-stimulated RAW264.7 macrophages.

### 3.8. Effects of Propolis Extracts on Nitric Oxide Production Before and After LPS Stimulation

**Figure 7:**
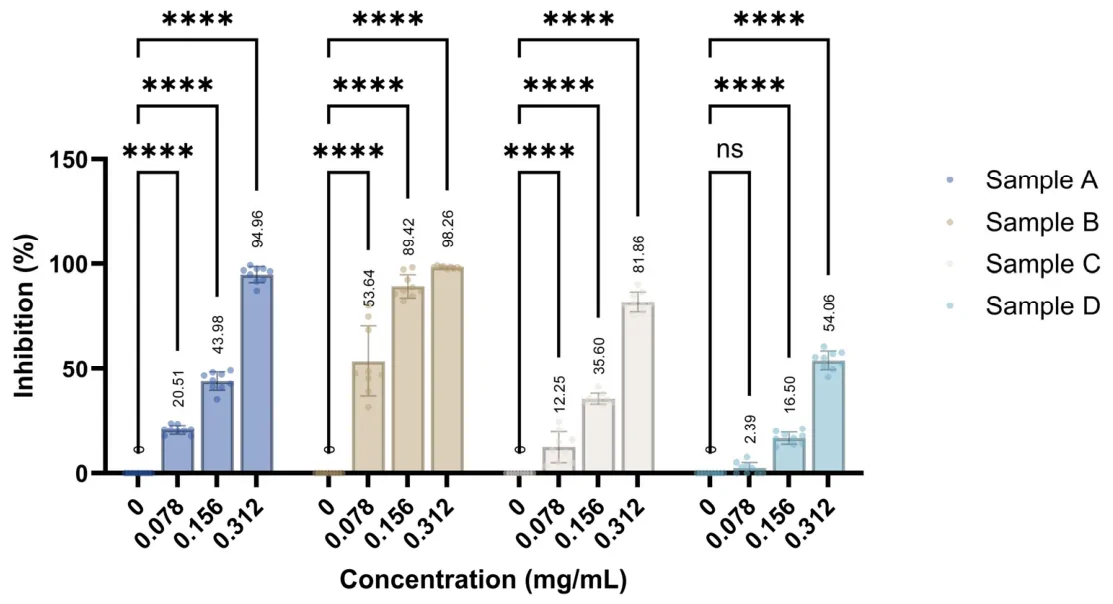
Inhibitory effects of propolis extracts on nitric oxide (NO) production in RAW 264.7 macrophages before LPS stimulation at concentrations of 0.078, 0.156, and 0.312 mg/mL. Asterisk indicates significant difference, **** (*p* ≤ 0.0001). ns indicates no significant difference.

The anti-inflammatory activity of propolis extracts was evaluated based on their inhibitory effects on nitric oxide (NO) production in RAW264.7 macrophages before and after lipopolysaccharide (LPS) stimulation. Nitric oxide production was measured by the Griess reaction after stimulation with 1 µg/mL LPS derived from *Escherichia coli* O111:B4. Before LPS stimulation, *T. pegdeni* stingless bee propolis extract (sample B) exhibited the strongest inhibitory activity, showing 89.42% and 98.26% inhibition of NO production at 0.156 and 0.312 mg/mL, respectively. The inhibitory activity gradually decreased with decreasing extract concentrations. *T. pegdeni* stingless bee propolis extract (sample A) also exhibited 94.96% inhibition of NO production at 0.312 mg/mL, whereas sample C and D showed 81.86% and 54.06% inhibition at the same concentration (Figure 7). These findings indicate that the propolis extracts differed in their ability to suppress LPS-induced NO production, with sample B showing the strongest inhibitory activity under the pre-treatment condition. Similar inhibitory effects of propolis on LPS-induced NO production have also been reported in macrophage models [34].

**Figure 8:**
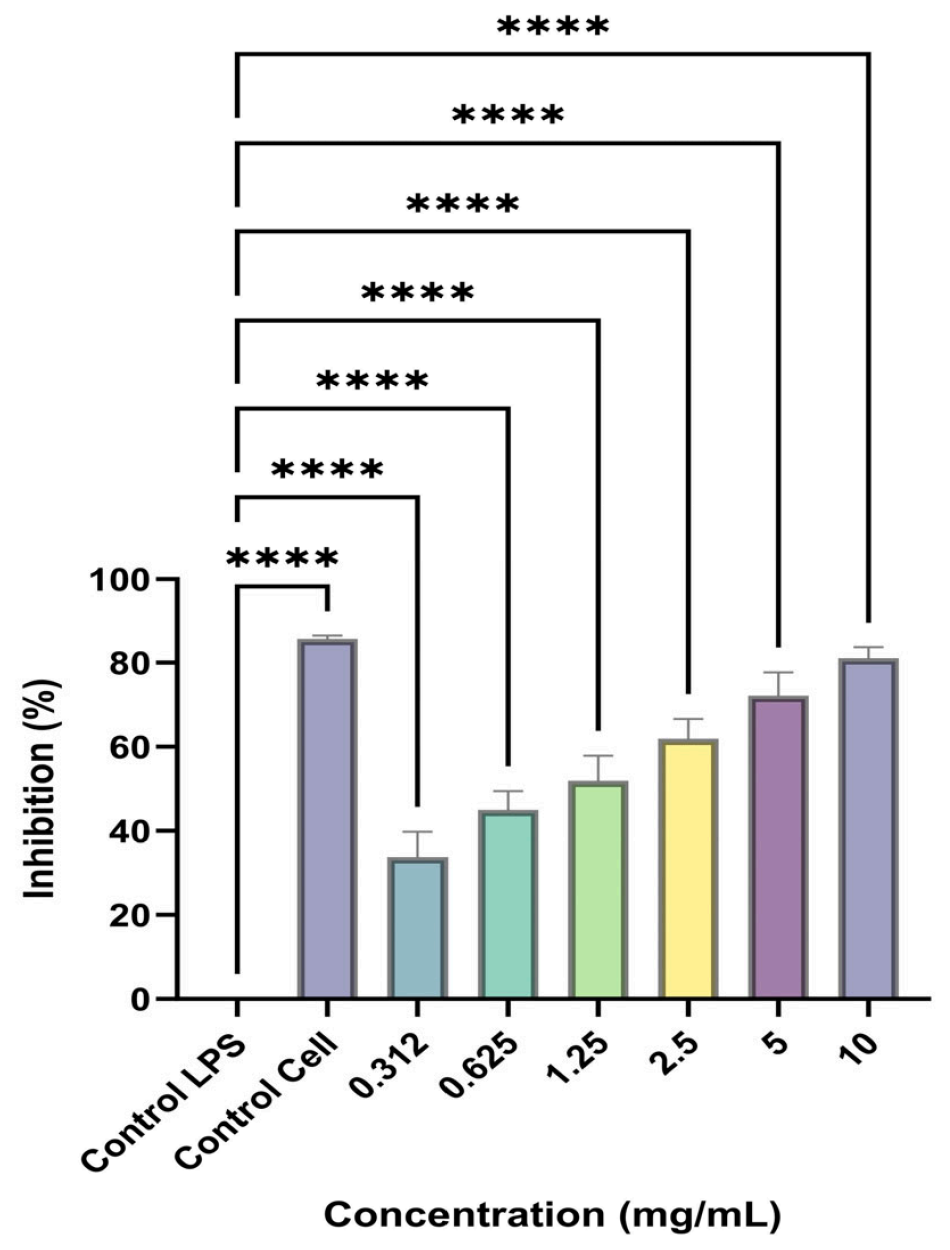
Effect of L-NAME at concentrations of 0.312–10 mg/mL on nitric oxide (NO) production in RAW 264.7 macrophages before LPS stimulation. Asterisk indicates significant difference, **** (*p* ≤ 0.0001).

The L-NAME positive control at non-toxic concentrations ranging from 0.312 to 10 mg/mL also showed inhibitory effects on NO production under the pre-treatment condition across the tested concentrations, supporting its use as a reference control for evaluating the NO inhibitory activity of the propolis extracts (Figure 8).

**Figure 9:**
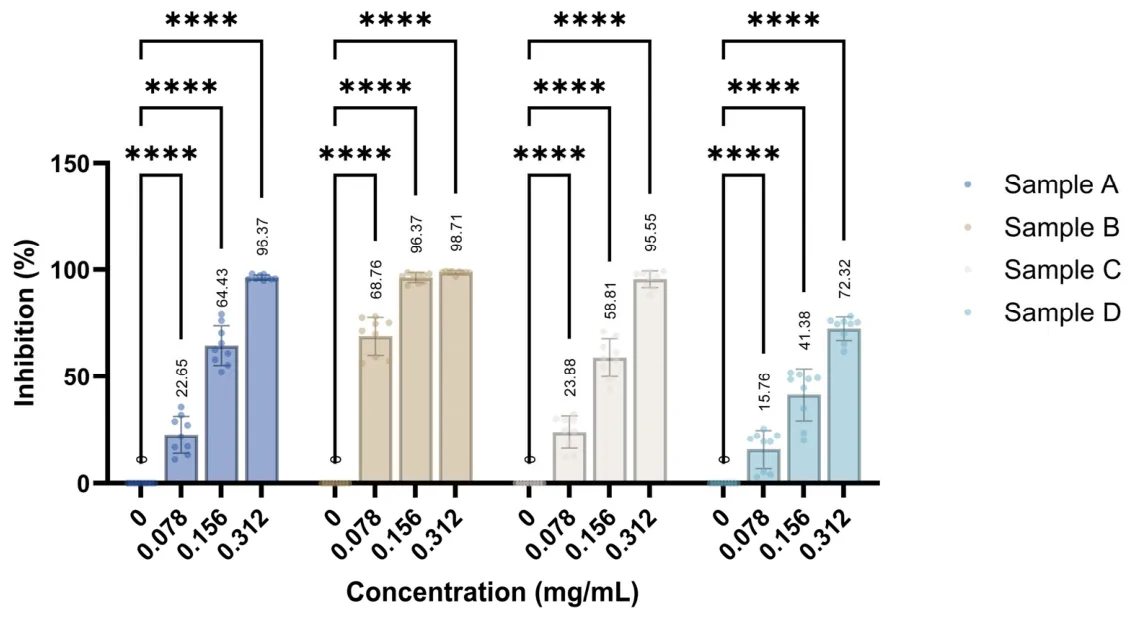
Inhibitory effects of propolis extracts on nitric oxide (NO) production in RAW 264.7 macrophages after LPS stimulation at concentrations of 0.078, 0.156, and 0.312 mg/mL. Asterisk indicates significant difference, **** (*p* ≤ 0.0001).

After LPS stimulation, *T. pegdeni* stingless bee propolis extract (sample B) exhibited the strongest inhibitory effect on NO production, followed by *T. pegdeni* stingless bee propolis extracts (samples A and C), whereas *T. laeviceps* stingless bee propolis extract (sample D) showed the lowest activity. Overall, all propolis extracts inhibited NO production in a concentration-dependent manner. These results demonstrated that the propolis extracts differed in their inhibitory effects on LPS-induced NO production. Based on the results of nitric oxide inhibition and cytotoxicity evaluation, *T. pegdeni* stingless bee propolis extracts (samples A and B) were selected for subsequent inflammatory gene expression analysis (Figure 9).

**Figure 10:**
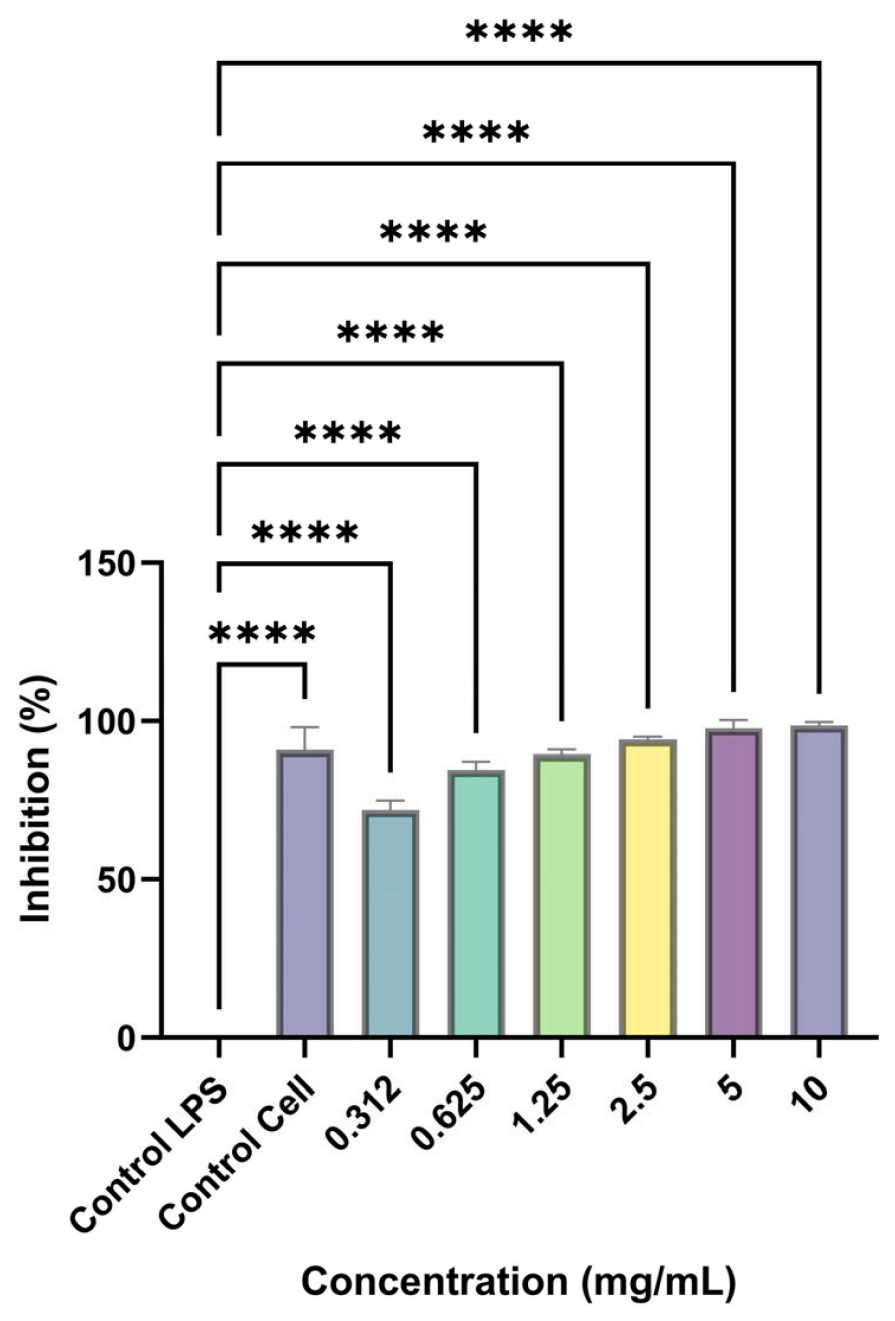
Effect of L-NAME at concentrations of 0.312–10 mg/mL on nitric oxide (NO) production in RAW 264.7 macrophages after LPS stimulation. Asterisk indicates significant difference, **** (*p* ≤ 0.0001).

L-NAME also significantly inhibited NO production under the post-treatment conditions, and higher concentrations of L-NAME generally showed greater NO inhibition (Figure 10).

### 3.9. Effects of Propolis Extracts on Inflammatory Gene Expression

**Figure 11:**
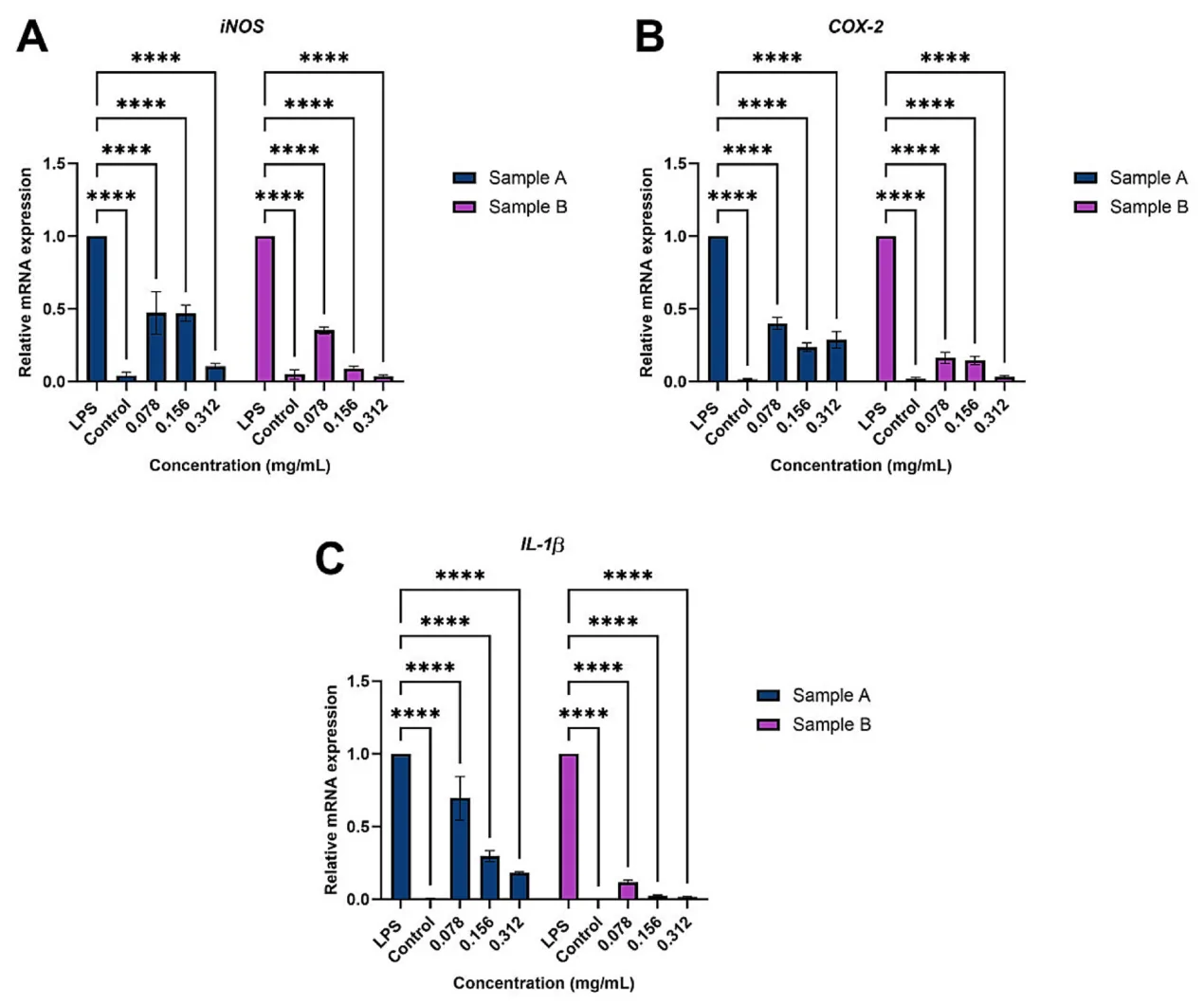
Effects of propolis extracts (samples A and B) on the mRNA expression levels of inflammatory genes in LPS-stimulated RAW264.7 macrophages after 24 h of treatment at concentrations of 0.078, 0.156, and 0.312 mg/mL (**A**) *iNOS*, (**B**) *COX*-*2*, and (**C**) *IL*-*1β*. Asterisk indicates significant difference, **** (*p* ≤ 0.0001).

The anti-inflammatory activity of propolis extracts was evaluated by analyzing the mRNA expression levels of inflammatory genes, including *iNOS*, *COX-2*, and *IL-1β*, in LPS-stimulated RAW264.7 macrophages using qRT-PCR. RAW 264.7 macrophages were exposed to lipopolysaccharide (LPS), followed by treatment with propolis extracts for 24 h before gene expression analysis. Following LPS stimulation, the expression levels of all inflammatory genes were significantly increased compared with the untreated control group. Treatment with propolis extracts significantly downregulated the expression of *iNOS*, *COX-2*, and *IL-1β* compared with the LPS-treated group (Figure 11).

*T. pegdeni* stingless bee propolis extract (samples A and B) exhibited inhibitory effects on inflammatory gene expression at concentrations of 0.078 to 0.312 mg/mL. Both propolis extracts markedly suppressed *iNOS* and *COX-2* expression at concentrations of 0.156 and 0.312 mg/mL. In addition, *IL-1β* expression was significantly reduced following treatment with both extracts, with *T. pegdeni* stingless bee propolis extract (sample B) showing stronger inhibitory activity at lower concentrations compared with *T. pegdeni* stingless bee propolis extract sample A (Figure 11). Overall, *T. pegdeni* stingless bee propolis extract (samples A and B) showed the most pronounced inhibitory effects on the expression of the inflammatory genes, particularly at 0.156 and 0.312 mg/mL. These results indicated that the propolis extracts exerted anti-inflammatory effects by reducing inflammatory gene expression in activated macrophages. Moreover, propolis has also been reported to inhibit NO production and inflammatory gene expression in LPS-stimulated RAW264.7 macrophages [38].

## 4. Discussion

The extraction method plays an important role in determining the extraction efficiency and quality of propolis extracts. Among various extraction approaches, maceration is widely employed as a conventional technique for propolis extraction due to its simple procedure and mild extraction conditions, which minimize the degradation of heat-sensitive bioactive compounds [39].

In the present study, 70% ethanol was selected as the extraction solvent due to its suitable polarity, which enables the extraction of a broad range of bioactive compounds, particularly phenolic compounds and flavonoids [40]. This selection is supported by previous studies demonstrating that 70% ethanol is an effective solvent for propolis extraction and facilitates the recovery of various phenolic and flavonoid compounds, including naringenin, kaempferol, and galangin, which are associated with considerable antioxidant properties [41,42].

However, although identical extraction conditions were applied to all samples, differences in extraction yields were still observed. These variations may be explained by differences in the amount and types of compounds present in each raw propolis sample that can be recovered during extraction. In the present study, *T*. *laeviceps* stingless bee propolis extract (sample D) exhibited the highest extraction yield, which may reflect a higher amount of extractable material in the raw propolis [43].

Furthermore, LC–MS analysis revealed a diverse chemical profile among the propolis extracts, reflecting the complex composition of propolis derived from different stingless bee species and geographical origins. Differences in chemical composition may be associated with variations in botanical sources of resin, geographical location, environmental conditions, and resin collection behaviour, all of which influence the types and abundance of bioactive constituents present in propolis [44,45]. Several classes of bioactive compounds were tentatively annotated, including flavonoids, phenolic acids, xanthones, terpenoids, triterpenoids, and curcuminoids. These compounds have been widely reported to be associated with the antioxidant, antimicrobial, and anti-inflammatory properties of propolis [46].

Propolis is a chemically complex natural product containing more than 300 identified constituents, including polyphenols (such as flavonoids, phenolic acids, and their esters), phenolic aldehydes, ketones, and various other compounds [47]. Among these constituents, phenolic compounds and flavonoids are considered important bioactive compounds and are widely recognized for their contribution to the biological activities of propolis, particularly its antioxidant activity [48]. Asem et al. [49] demonstrated that apositive correlation between total phenolic and flavonoid contents and the antioxidant activity of Malaysian stingless bee propolis extracts. Similarly, Abduh et al. [50] reported that the total phenolic and flavonoid contents of *T. laeviceps* propolis were positively associated with its antioxidant potential. Among all of the propolis samples analyzed, *T*. *laeviceps* stingless bee propolis extract (sample D) showed the highest total phenolic and flavonoid contents. The observed variations in TPC and TFC among the propolis samples may be attributed to differences in the chemical composition of raw propolis, which is strongly influenced by botanical origin and environmental factors [44,45]. In particular, the botanical origin of resin sources is considered one of the key factors determining the chemical composition of propolis, as different plant sources contain diverse profiles of phenolic compounds, flavonoids, and other secondary metabolites [51].

In the present study, the stingless bee propolis samples were collected from different environments, including a mixed fruit orchard (sample A), a mango-dominant orchard (samples B and D), and a herbal garden (sample C). The differences in surrounding vegetation among these collection sites may influence the availability of potential resin sources and consequently contribute to variations in the phenolic and flavonoid contents of the obtained propolis extracts. This finding is also supported by previous studies showing that propolis collected from different plant sources and geographical regions exhibits distinct chemical profiles and characteristic compounds, such as caffeic acid esters in poplar-type propolis, artepillin C in Brazilian propolis, and polyisoprenylated benzophenones in Cuban propolis. These findings further highlight the important role of botanical origin in determining the chemical diversity of propolis [52].

Interestingly, although *T. pegdeni* stingless bee propolis (samples B and D) were collected from the same mango-dominant orchard, they were obtained from colonies of different stingless bee species (*T. pegdeni* and *T. laeviceps*). The distinct differences in TPC and TFC observed between these samples may be associated with multiple factors, including botanical origin, environmental conditions, and resin collection behaviour, and therefore cannot be attributed specifically to bee species based on the present sampling design. This observation is consistent with previous reports indicating that stingless bee propolis exhibits high chemical diversity and complexity, which may be influenced by differences in botanical sources and environmental conditions [53]. Similarly, Popova et al. [54]. reported that propolis from different stingless bee species exhibited distinct chemical profiles, highlighting the potential contribution of bee species and resin collection behaviour to propolis chemical diversity. However, the relative contribution of these factors cannot be distinguished in the present study because of the limited sampling design.

Although *T*. *laeviceps* stingless bee propolis extract (sample D) exhibited the highest total phenolic and flavonoid contents, *T. pegdeni* stingless bee propolis extract (sample A) demonstrated the strongest antioxidant activity in the DPPH, ABTS, and FRAP assays. Previous studies have generally reported a positive correlation between total phenolic and flavonoid contents and the antioxidant activity of propolis [55,56]. However, the present findings suggest that higher total phenolic and flavonoid contents did not necessarily correspond to stronger antioxidant activity. Similarly, Idrobo et al. [57] reported that total phenolic content alone did not fully explain the antioxidant activity of propolis extracts, suggesting that differences in the composition of individual phenolic compounds may also contribute to antioxidant capacity. One possible explanation is that the Folin–Ciocalteu and aluminum chloride colorimetric assays determine only the total phenolic and flavonoid contents and do not provide information on the individual phenolic compounds present in each extract [58,59].

Differences in the phenolic composition of the extracts may partly explain the variation in antioxidant activity observed in this study. The antioxidant activity of phenolic compounds is influenced not only by their concentration but also by their chemical structure, especially the number and position of hydroxyl groups, which can affect their ability to donate hydrogen atoms or electrons and scavenge free radicals [60]. In addition, propolis is a chemically complex natural product containing various bioactive constituents such as phenolic compounds and flavonoids, along with terpenoids, aromatic acids, steroids, and other secondary metabolites. These compounds may also contribute to the overall antioxidant activity of propolis [51,61]. Despite the differences in antioxidant capacity among the extracts, all propolis samples demonstrated considerable antioxidant activity. Therefore, the differences in antioxidant activity observed among the propolis extracts are likely associated with variations in their overall chemical composition. Such variations may arise from differences in botanical origin, stingless bee species, geographical location, environmental conditions, and collection time. Similar observations have been reported in previous studies, demonstrating that propolis collected from different geographical regions and plant sources exhibits distinct chemical compositions and antioxidant activities [62,63].

Stingless bee propolis has attracted considerable attention because of its remarkable antibacterial activity against various pathogenic bacteria [64]. In the present study, different propolis extracts showed different antibacterial activities against the tested bacterial species. Sample A showed antibacterial activity against all bacterial species tested, with inhibition zone diameters ranging from 7.00 to 14.00 mm, whereas the remaining propolis extracts inhibited only some of the tested bacteria. Previous studies have demonstrated that propolis exhibits antibacterial activity against a wide range of pathogenic bacteria.

Malaysian propolis extracts have been reported to inhibit both Gram-positive and Gram-negative bacteria, including *Bacillus subtilis*, *Staphylococcus aureus, Escherichia coli*, and *Salmonella* spp. [65]. Likewise, Turkish propolis exhibited dose-dependent antibacterial activity against several *Streptococcus* species [66]. In addition, a recent review summarized extensive evidence supporting the broad-spectrum antimicrobial activity of stingless bee propolis against numerous pathogenic microorganisms [67], with Gram-positive bacteria generally showing greater susceptibility than Gram-negative bacteria. Similarly, the agar disc diffusion assay in the present study showed that the propolis extracts exhibited stronger antibacterial activity against Gram-positive bacteria than against *Ps*. *aeruginosa*. This difference may be attributed to variations in bacterial cell wall structure. Gram-negative bacteria have an outer membrane that serves as an additional protective barrier, making it more difficult for bioactive compounds, such as polyphenols, to enter the bacterial cell. In contrast, Gram-positive bacteria lack an outer membrane, allowing these compounds to interact more directly with the peptidoglycan layer and exert antibacterial effects [68]. The MIC and MBC results further supported the findings of the agar disc diffusion assay, with Gram-positive bacteria generally exhibiting lower MIC and MBC values than Gram-negative bacteria. These results further support the role of the Gram-negative outer membrane as a barrier that limits the antibacterial activity of bioactive compounds.

Interestingly, some propolis extracts that showed no visible inhibition zones in the agar disc diffusion assay were still able to inhibit bacterial growth in the broth dilution assay. Although *T. pegdeni* stingless bee propolis extract (sample B) produced no inhibition zones against *S. aureus*, *S. epidermidis*, and *C. acnes*, growth inhibition was observed in the MIC assay, with MIC values of 7.81 mg/mL. Similar results were also observed for *T*. *laeviceps* stingless bee propolis extract (sample D), which inhibited the growth of several bacterial species despite the absence of visible inhibition zones in the agar disc diffusion assay. These differences may be explained by the different principles of the two assays. The agar disc diffusion assay is a diffusion-based method, in which the observed inhibition zone depends on the ability of bioactive compounds to diffuse through the agar medium. Therefore, the inhibition zone size can be affected by the physicochemical properties of the extracts and their bioactive constituents, including solubility and diffusion characteristics [69]. In contrast, the broth dilution method determines antibacterial activity based on the direct interaction between bacterial cells and the tested extracts at defined concentrations, providing quantitative MIC values. Therefore, MIC determination may better reflect the antibacterial potential of extracts containing bioactive compounds with limited agar diffusion ability [70]. Previous studies have also indicated that differences between diffusion and dilution methods can lead to variations in the observed antibacterial activity [71]. This may explain the differences observed for *T. pegdeni* stingless bee propolis extract (sample B) and *T*. *laeviceps* stingless bee propolis extract (sample D), which showed antibacterial activity in the broth dilution assay despite the absence of visible inhibition zones in the agar disc diffusion assay.

Lower MIC and MBC values indicate greater antibacterial potency, as lower concentrations of the extract are required to inhibit or kill bacterial cells [72]. In the present study, *T. pegdeni* stingless bee propolis extract (sample B) exhibited relatively low MIC and MBC values against several Gram-positive bacteria, suggesting the presence of potent antibacterial constituents. The antibacterial activity of propolis extracts is likely associated with their complex chemical composition, particularly phenolic compounds and flavonoids, which can exert antimicrobial effects through several mechanisms. Previous studies on Thai stingless bee propolis have identified several antibacterial constituents, including phenolic acids (e.g., *p*-coumaric acid and caffeic acid), flavonoids (e.g., pinocembrin, chrysin, galangin, and quercetin), and terpenoids, which are considered major contributors to the antibacterial activity of propolis [10].

Among these compounds, *p*-coumaric acid, one of the major phenolic acids commonly found in propolis, has been reported to increase bacterial membrane permeability, leading to leakage of intracellular contents, and to interfere with DNA replication, transcription, and gene expression through binding to bacterial genomic DNA [73]. Likewise, pinocembrin, a major flavonoid in propolis, has been shown to disrupt bacterial membrane integrity and membrane permeability, while also suppressing proteins involved in protein synthesis, energy metabolism, and iron uptake, thereby inhibiting bacterial growth [74]. Chrysin, a major flavonoid found in propolis, has been reported to exert antibacterial activity through several mechanisms, including altering bacterial membrane potential, damaging the outer membrane, disrupting membrane integrity, and inhibiting biofilm formation [75]. Collectively, these findings suggest that the antibacterial activity of propolis is mediated through multiple complementary mechanisms, including membrane disruption, leakage of intracellular contents, interference with nucleic acid and protein synthesis, inhibition of energy metabolism, and suppression of biofilm formation. These properties may contribute to the potential use of propolis in oral healthcare. Previous studies have reported the potential use of propolis in the prevention and management of oral diseases, including dental caries, gingivitis, and periodontitis, due to its antimicrobial and anti-inflammatory properties [76].

LC–MS analysis revealed differences in the chemical profiles among the propolis extracts. Several bioactive compounds, including quercetin and *p*-coumaric acid, were detected at relatively higher abundances in *T. pegdeni* stingless bee propolis extracts (samples A and B) than in the other propolis samples. Quercetin, one of the major flavonoids commonly identified in propolis, has been widely reported to inhibit a broad range of Gram-positive and Gram-negative bacteria by disrupting bacterial membrane integrity, inhibiting nucleic acid and protein synthesis, suppressing virulence factors, and preventing biofilm formation. Likewise, *p*-coumaric acid, a hydroxycinnamic phenolic acid commonly found in propolis, has been shown to impair bacterial membrane integrity, increase membrane permeability, and promote leakage of intracellular constituents, thereby inhibiting bacterial growth [77,78,79]. These findings suggest that quercetin and *p*-coumaric acid may contribute to the antibacterial activity observed in *T. pegdeni* stingless bee propolis extract (samples A and B). However, as these compounds were not individually isolated or quantified in the present study, their specific contributions to the observed antibacterial activity remain unclear. The antibacterial activity of propolis may instead result from the combined or synergistic effects of multiple constituents rather than from a single compound.

The cytotoxic effects of propolis extracts on RAW264.7 macrophages were evaluated to select suitable concentrations for the anti-inflammatory assays. All extracts showed cell viability above 70% at the tested concentrations. Based on these results, 0.078, 0.156, and 0.312 mg/mL of propolis extracts were selected for subsequent evaluation of NO production and inflammation-related gene expression in LPS-stimulated RAW264.7 macrophages.

Several studies have demonstrated that propolis exhibits anti-inflammatory activity by suppressing inflammatory mediators in activated macrophages [22,80,81]. Nitric oxide (NO) is an important mediator of inflammation that is produced by activated macrophages. Excessive NO production can contribute to inflammatory responses [82]. In the present study, LPS stimulation increased NO production in RAW264.7 macrophages, whereas treatment with propolis extracts significantly reduced NO production in a concentration-dependent manner. Among the propolis extracts, samples A and B exhibited the strongest inhibitory activities. Sample B showed the greatest reduction in NO production at concentrations of 0.156 and 0.312 mg/mL after LPS stimulation, while sample A also exhibited marked inhibitory activity, particularly at 0.312 mg/mL. These findings suggest that both extracts possess anti-inflammatory potential by inhibiting LPS-induced NO production. Similar anti-inflammatory effects have also been reported for *Lepidotrigona* propolis, which significantly suppressed NO production in LPS-stimulated RAW264.7 macrophages [83]. Previous studies have suggested that the anti-inflammatory activity of propolis is associated with the suppression of *iNOS* expression, leading to reduced production of inflammatory mediators such as NO. This effect has been attributed to bioactive polyphenolic compounds, including caffeic acid, which suppress the *NF-κB* and p38 MAPK signalling pathways [81,84]. Based on their favourable cell viability and strong NO inhibitory activities, samples A and B were selected for further investigation of inflammation-related gene expression.

Gene expression analysis showed that both *T. pegdeni* stingless bee propolis extracts (samples A and B) significantly reduced the expression of *iNOS*, *COX-2*, and *IL-1β* in LPS-stimulated RAW264.7 macrophages in a concentration-dependent manner. Sample B showed stronger inhibitory effects than sample A, especially at 0.312 mg/mL. These results agree with previous studies showing that propolis extracts can reduce LPS-induced inflammation in macrophages by decreasing NO production and the expression of inflammatory genes, including *COX-2* and *IL-1β* [85]. In the present study, the reduction in NO production was consistent with the decreased *iNOS* expression, suggesting that propolis extracts may inhibit NO production by suppressing *iNOS* expression. The reduced expression of *COX-2* and *IL-1β* further indicates that propolis extracts can regulate several inflammatory responses induced by LPS.

Similar anti-inflammatory effects have been reported for Brazilian green propolis extracts. Machado et al. [86] found that aqueous extracts of green propolis reduced inflammation in an LPS-induced inflammation model by decreasing the accumulation of macrophages and neutrophils and regulating cytokine production, including reduced *TNF-α* and *IL-6* levels and increased *IL-10* and *TGF-β* levels. Similarly, Zamarrenho et al. [87] found that the different Brazilian green propolis extract formulations affected cytokine production in macrophages by regulating pro-inflammatory and anti-inflammatory cytokines, such as *IL-6*, *TNF-α*, and *IL-10*. These findings, together with our results, suggest that propolis extracts may reduce inflammation by affecting multiple inflammatory mediators. Zulhendri et al. [83] the anti-inflammatory activity of propolis is associated with the regulation of multiple inflammatory signalling pathways. Since inflammation is controlled by complex signalling, excessive activation of these pathways can lead to increased production of inflammatory mediators and prolonged inflammatory responses. Propolis has been reported to regulate several key inflammatory components, including TLR4-related signalling molecules, MyD88, IRAK4, TRIF, NLRP inflammasomes, and *NF-κB*, resulting in the reduced production of pro-inflammatory cytokines such as *IL-1β*, *IL-6*, and *TNF-α*. These regulatory effects may contribute to the suppression of inflammatory gene expressions involved in macrophage activation. Therefore, the decreased expression of *iNOS*, *COX-2*, and *IL-1β* observed in the present study, together with the reduction in NO production, suggests that propolis extracts may reduce LPS-induced inflammatory responses through the modulation of inflammatory signalling processes. The potential application of propolis in inflammatory conditions has also been explored in multi-component approaches. A recent review proposed a formulation containing propolis, *Cissus quadrangularis*, *Boswellia serrata*, and palmitoylethanolamide to target multiple pathological processes associated with osteoarthritis, including inflammation, oxidative stress, cartilage degradation, and pain [88].

The anti-inflammatory effects observed in this study may be associated with the complex mixture of bioactive constituents present in the propolis extracts, including phenolic acids, flavonoids, and other phenolic compounds tentatively annotated by LC–MS analysis. Previous studies have shown that many of these compounds possess anti-inflammatory properties by reducing the production of inflammatory mediators and modulating inflammation-related signalling pathways [81,89]. In the present study, several bioactive compounds identified in *T. pegdeni* stingless bee propolis extract (samples A and B) have previously been reported to exhibit anti-inflammatory properties. Mangiferin has been shown to inhibit *iNOS* expression, reduce ROS production, suppress NLRP3 inflammasome activation, and decrease *IL-1β* production [90]. Rosmarinic acid has also been reported to suppress inflammatory mediators, such as *iNOS*, *COX-2*, *IL-1β*, *TNF-α*, and *NF-κB* [91]. In addition, flavonoids such as velutin and quercetin, as well as curcumin, have been shown to reduce NO production and suppress inflammatory mediators through the regulation of signalling pathways involved in inflammation. These reported activities agree with the present findings, in which samples A and B reduced NO production and downregulated the expression of *iNOS*, *COX-2*, and *IL-1β* in LPS-stimulated RAW264.7 macrophages. Therefore, the anti-inflammatory activity observed in this study may be associated with the combined effects of multiple bioactive constituents rather than a single constituent [92,93,94,95]. A limitation of this study is that the biological activities of the propolis extracts cannot be directly linked to any specific compound detected by LC–MS. Several compounds with previously reported biological activities were tentatively annotated based on their mass spectral characteristics and comparisons with the available databases and literature. However, individual compounds were not isolated or purified, and bioassay-guided fractionation or correlation analysis was not performed. Therefore, these compounds should be regarded as potential contributors to the observed activities rather than confirmed active constituents. The antimicrobial and anti-inflammatory effects may reflect the combined or synergistic actions of multiple compounds present in the complex propolis matrix. Further studies involving fractionation, isolation and purification of individual compounds, quantitative analysis, and confirmation using authentic standards are needed to determine the contribution of specific compounds to the biological activities of Thai stingless bee propolis.

## 5. Conclusions

This study showed that Thai stingless bee propolis extracts contain diverse chemical constituents and exhibit various biological activities. LC–MS analysis tentatively annotated several groups of compounds, including phenolic acids, flavonoids, xanthones, terpenoids, triterpenoids, and curcuminoids. Some of these compounds have previously been reported to possess antioxidant, antibacterial, or anti-inflammatory properties and may be associated with the activities observed in the present study. Differences in chemical composition and biological activities among the individual propolis extracts may reflect variation in their botanical and environmental origins.

All stingless bee propolis extracts exhibited antioxidant and antibacterial activities against the tested bacteria and showed anti-inflammatory effects, as indicated by reduction in NO production and downregulated *iNOS*, *COX-2*, and *IL-1β* gene expression in LPS-stimulated RAW264.7 macrophages. These activities may result from the combined effects of multiple constituents in the propolis extracts rather than from a single compound. However, the contribution of individual LC–MS-annotated compounds to the observed biological activities could not be confirmed in the present study. Further studies involving fractionation, isolation and purification of individual compounds, quantitative analysis, and validation using authentic standards are needed to clarify their contributions to the biological activities of Thai stingless bee propolis. Overall, Thai stingless bee propolis shows potential as a natural source of compounds with antioxidant, antimicrobial, and anti-inflammatory activities.

## Figures and Tables

**Table 1:** Sampling locations and geographic coordinates of Thai stingless bee propolis samples.

Sample	Stingless Bee Species	Province	Sampling Location	Latitude	Longitude
A	*T*. *pegdeni*	Chumphon	Khun Krathing Subdistrict, Mueang Chumphon District	10.4951° N	99.1372° E
B	*T*. *pegdeni*	Chiang Mai	Ban Pa Chu, Makham Luang Subdistrict, San Pa Tong District	18.5990° N	98.9016° E
C	*T*. *pegdeni*	Sa Kaeo	Exact sampling site unavailable	Not available	Not available
D	*T*. *laeviceps*	Chiang Mai	Ban Pa Chu, Makham Luang Subdistrict, San Pa Tong District	18.5990° N	98.9016° E

**Table 2:** Gradient elution programme used for LC–MS/MS analysis of propolis extracts.

Time (min)	Mobile Phase A (%)	Mobile Phase B (%)
0.0	100	0
0.5	100	0
10.5	45	55
12.5	25	75
14.0	0	100
17.0	0	100
17.5	100	0
20.0	100	0

**Table 3:** Percentage yields of propolis extracts.

Sample	Stingless Bee Species	Collection Site	Extraction Yield (%)
A	*T. pegdeni*	Chumphon	18.63
B	*T. pegdeni*	Chiang Mai	21.97
C	*T. pegdeni*	Sa Kaeo	25.77
D	*T. laeviceps*	Chiang Mai	28.51

**Table 4:** Total phenolic and flavonoid contents of the propolis extracts.

Sample	Stingless Bee Species	Total Phenolic Content (mg GAE/g Extract)	Total Flavonoid Content (mg QUE/g Extract)
A	*T*. *pegdeni*	9.29 ± 0.25 ^a^	1.57 ± 0.66 ^a^
B	*T*. *pegdeni*	4.39 ± 0.27 ^a^	45.02 ± 1.50 ^b^
C	*T*. *pegdeni*	5.98 ± 0.85 ^a^	7.93 ± 0.12 ^a^
D	*T*. *laeviceps*	29.59 ± 0.28 ^b^	73.95 ± 4.62 ^c^

Different letters (a–c) indicate statistically significant differences among the values, with differences considered significant at *p* < 0.05.

**Table 5:** Antioxidant activities of propolis extracts determined using the DPPH, ABTS, and FRAP assays.

Sample	Stingless Bee Species	DPPH		ABTS		FRAP
		IC_50_ (mg/mL)	Antioxidant Activity (mg GAE/g Extract)	IC_50_ (mg/mL)	Antioxidant Activity (mg TEAC/g Extract)	Antioxidant Activity (mg FeSO_4_/g Extract)
A	*T*. *pegdeni*	0.80 ± 0.02 ^a^	7.08 ± 0.18 ^b^	3.64 ± 0.00 ^a^	75.64 ± 3.07 ^b^	48.54 ± 0.48 ^d^
B	*T*. *pegdeni*	2.55 ± 0.46 ^c^	2.25 ± 0.42 ^a^	5.18 ± 0.59 ^bc^	53.41 ± 2.10 ^a^	17.51 ± 1.58 ^a^
C	*T*. *pegdeni*	1.06 ± 0.13 ^ab^	5.43 ± 0.71 ^b^	3.84 ± 0.22 ^ab^	71.83 ± 0.07 ^b^	21.63 ± 1.74 ^b^
D	*T*. *laeviceps*	1.74 ± 0.08 ^b^	3.25 ± 0.13 ^a^	5.36 ± 0.21 ^c^	51.44 ± 0.72 ^a^	35.45 ± 1.40 ^c^

Statistical significance was denoted by different letters (a–d) indicating a significant difference (*p* < 0.05). Values sharing the same letter (e.g., ab and bc) were not significantly different.

**Table 6:** Antibacterial activity of propolis extracts against skin pathogenic bacteria determined by the agar disc diffusion assay.

Sample (500 mg/mL)	Stingless Bee Species	Inhibition Zone Diameter (mm)					
		*S*. *aureus*	*S*. *epidermidis*	MRSA	*Ps*. *aeruginosa*	*M*. *luteus*	*C*. *acnes*
A	*T*. *pegdeni*	8.17 ± 0.29 ^b^	8.73 ± 0.25 ^b^	10.00 ± 1.00 ^c^	7.00 ± 0.00 ^b^	12.33 ± 0.58 ^c^	14.00 ± 0.00 ^c^
B	*T*. *pegdeni*	0 ^a^	0 ^a^	7.83 ± 0.29 ^b^	0 ^a^	0 ^a^	0 ^a^
C	*T*. *pegdeni*	9.00 ± 0.00 ^c^	9.83 ± 0.29 ^c^	9.83 ± 0.29 ^c^	0 ^a^	11.33 ± 0.58 ^c^	10.67 ± 0.58 ^b^
D	*T*. *laeviceps*	0 ^a^	0 ^a^	0 ^a^	0 ^a^	8.67 ± 0.58 ^b^	0 ^a^
Gentamicin (1 mg/mL)		23.00 ± 0.00 ^d^	23.33 ± 0.58 ^d^	-	31.50 ± 0.50 ^c^	31.67 ± 1.53 ^d^	26.33 ± 1.15 ^d^
Vancomycin (1 mg/mL)		-	-	20.00 ± 0.00 ^d^	-	-	-

Data are expressed as mean ± SD (*n* = 3). Inhibition zones (mm) were measured by the agar disc diffusion method using propolis extracts at a concentration of 500 mg/mL. Different letters (a–d) within each bacterial strain indicate statistically significant differences (*p* < 0.05), as determined by one-way ANOVA followed by Tukey’s HSD test. Gentamicin and vancomycin, each at 1 mg/mL, served as positive controls.

**Table 7:** MIC and MBC values of propolis extracts against skin pathogenic bacteria.

Sample	Stingless Bee Species	Concentration of Propolis Extracts (mg/mL)											
		*S*. *aureus*		*S*. *epidermidis*		*MRSA*		*Ps*. *aeruginosa*		*M*. *luteus*		*C*. *acnes*	
		MIC	MBC	MIC	MBC	MIC	MBC	MIC	MBC	MIC	MBC	MIC	MBC
A	*T* *. pegdeni*	3.90	3.90	15.62	15.62	31.25	31.25	125	125	1.95	1.95	62.5	62.5
B	*T* *. pegdeni*	7.81	7.81	7.81	7.81	15.63	15.63	125	125	1.95	1.95	7.81	7.81
C	*T* *. pegdeni*	7.81	7.81	7.81	7.81	125	125	125	125	1.95	1.95	3.90	3.90
D	*T* *. laeviceps*	15.62	15.62	31.25	31.25	31.25	31.25	125	125	0.97	0.97	62.5	62.5
Gentamicin		0.125	0.125	0.0019	0.0019	-	-	0.0098	0.0098	0.0078	0.0078	0.0312	0.0312
Vancomycin		-	-	-	-	0.125	0.125	-	-	-	-		-

## Data Availability

Data are contained within the article.

## Abbreviations

The following abbreviations are used in this manuscript:

ABTS	2,20-azinobis-(3-ethylbenzothiazolin-6-sulfonic acid
DPPH	2,2-diphenyl-1-picrylhydrazil
FRAP	ferric reducing antioxidant power
GAE	gallic acid equivalent
TEAC	trolox equivalent antioxidant capacity
LC-MS	liquid chromatography–mass spectrometry
MIC	minimum inhibitory concentration
MBC	minimum bactericidal concentration
DMEM	Dulbecco’s modified Eagle’s medium
iNOS	Inducible nitric oxide synthase
COX-2	Cyclooxygenase-2
IL-1β	Interleukin-1 beta
